# Novel technique with bladder peritoneum to prevent empty pelvic syndrome after laparoscopic pelvic exenteration for gynecologic malignancies: Three case reports

**DOI:** 10.1097/MD.0000000000028200

**Published:** 2021-12-10

**Authors:** Yiran Wang, Ping Wang

**Affiliations:** a Department of Gynecology and Obstetrics, West China Second University Hospital, Sichuan University, Chengdu, Sichuan, PR China; b Key Laboratory of Birth Defects and Related Diseases of Women and Children (Sichuan University), Ministry of Education, Chengdu, Sichuan, PR China.

**Keywords:** bladder peritoneum, empty pelvic syndrome, gynecologic malignancy, laparoscopy, pelvic exenteration

## Abstract

**Rationale:**

Pelvic exenteration (PE) is a radical surgical procedure for treating locally recurrent or uncontrolled pelvic malignancies. The consequent postoperative pelvic dead space presents a challenge to extirpative surgeons. Many methods have been utilized for pelvic floor reconstruction to reduce related postoperative complications, however, none of them have been widely accepted.

**Patient concerns:**

Here, we report 3 cases of patients who underwent PE. Case 1 was a 36-year-old woman who presented to our hospital with abnormal vaginal bleeding. Case 2 was a 50-year-old woman with recurrence of stage IIB squamous cell carcinoma of the cervix. Case 3 was a 54-year-old woman with uncontrolled stage IIB adenocarcinoma of the cervix. The last 2 patients were both treated with radiotherapy and chemotherapy previously.

**Diagnosis:**

Biopsy results revealed adenocarcinoma of the vagina, squamous cell carcinoma of the cervix, and adenocarcinoma of the cervix in Case 1, 2, and 3 respectively.

**Interventions:**

We describe a safe and effective approach that employs the preservation of the bladder peritoneum to eliminate the pelvic dead space following laparoscopic PE, with or without partial utilization of the greater omentum.

**Outcomes:**

Three patients with gynecologic cancer underwent this operation and developed no intraoperative or postoperative complications.

**Conclusion:**

Our experience suggests that laparoscopic PE using the bladder peritoneal barrier to cover the denuded pelvic cavity is a reasonable choice to decrease the risk of empty pelvic syndrome.

## 1 Introduction

Pelvic exenteration (PE) was first described by Alexander Brunschwig in 1948 for the palliative surgical treatment of recurrent gynecologic malignancies,^[1]^ but subsequently became the only curative option for locally advanced and recurrent pelvic cancers after previous radiotherapy, chemotherapy, or surgery.^[2]^ This ultraradical surgery, involving complete en bloc removal of malignant lesions and pelvic viscera, is associated with a reported morbidity ranging from 32% to 84%.^[3]^ A substantial proportion of the severe postoperative morbidity arises from empty pelvic syndrome, which can be defined as various symptoms due to the pelvic dead space after PE, such as abscess formation, bowel obstruction, hematoma, fistulization, herniation, persistent discharge, and wound dehiscence.^[4]^ Multiple methods and materials have been utilized to avoid these complications including reconstructive procedures involving the utilization of the greater omentum,^[5]^ mammary prosthesis,^[6]^ myocutaneous flaps,^[7–10]^ dura mater allografts,^[11]^ or using a degradable mesh,^[4]^ to eliminate the pelvic defect. However, so far, the search for an ideal solution continues.

We report 3 cases in which the bladder peritoneum was preserved and used to close the pelvic cavity following laparoscopic PE for gynecologic malignancies. The types of laparoscopic PE performed were total pelvic exenteration (TPE) or anterior pelvic exenteration (APE), depending on the case. All 3 patients underwent operations with our novel technique that uses the bladder peritoneum to act as a biological partition to prevent empty pelvic syndrome by laparoscopy. Meanwhile, a portion of the greater omentum was employed to fill the void following TPE, because of its bulk. The basic characteristics and treatment information of the 3 patients are summarized in Table 1.

**Table 1:** Patients’ basic characteristics and therapy.

	Case 1	Case 2	Case 3
Gender	Female	Female	Female
Age	36	50	54
BMI (kg/m^2^)	23.7	21.5	19.8
Preoperative diagnosis	Adenocarcinoma of the vagina (bladder invasion)	Recurrence of stage IIB cervical squamous cell carcinoma	Uncontrolled stage IIB cervical adenocarcinoma
Previous treatment	None	RT+CT	RT+CT
Type of exenteration	Anterior	Anterior	Total
Urinary diversion	Ileal conduit	Ureterocutaneostomy	Ureterocutaneostomy
Intestinal reconstruction	End-to-end ileoileostomy	None	Sigmoidostomy
Vaginal reconstruction	Partial bladder peritoneum + rectouterine peritoneum	None	None
Pelvic floor reconstruction	Bladder peritoneum	Bladder peritoneum	Bladder peritoneum + a portion of the greater omentum
Operative time (min)	300	320	400
Blood loss (mL)	400	200	600
Pathology	Clear cell adenocarcinoma of the vagina	Poorly differentiated squamous cell carcinoma of the cervix	Poorly differentiated endometrioid adenocarcinoma of the cervix
FIGO stage	IVA	IIIC1	IVA
Resection margins	Microscopically negative (R0)	Microscopically negative (R0)	Microscopically negative (R0)
Postoperative hospitalization (d)	10	11	14
Postoperative complication	None	None	None
Follow-up time (mo)	10	14	6
Last follow-up status	Alive	Alive	Alive

## 2 Methods

### 2.1 Ethical approval and patient consent

This study was approved by the medical ethics committee of West China Second Hospital of Sichuan University (2021 Medical scientific research for ethical approval number: 89). Informed written consents were obtained from the patients for the purpose of publication.

### 2.2 Surgical technique

**Figure 1:**
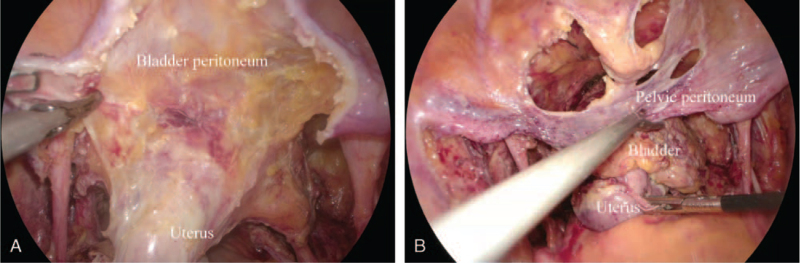
(A) The dissection began by incising the anterior lobe of the broad ligament and was continued anteriorly in the uterovesical peritoneal reflection. (B) The bladder peritoneum was peeled off the bladder.

The procedure was performed following a standardized laparoscopic PE technique,^[12,13]^ except that the peritoneum covering the surface of the bladder was preserved during cystectomy and was used for closure of the pelvic cavity. The dissection began by incising the anterior lobe of the broad ligament and was continued anteriorly in the uterovesical peritoneal reflection (Fig. 1A). The bladder peritoneum was peeled off the bladder (Fig. 1B), and the Retzius space was dissected. Other steps were the same as the conventional extirpative procedure. During the reconstructive procedure, the free edge of the preserved bladder peritoneum was sutured to the rectal fascia in the APE or the pelvic floor fascia in the TPE. The patients were administered antibiotics during the surgery to prevent infection for Class II incision.

## 3 Case report

### 3.1 Case 1

A 36-year-old woman was admitted to our department with abnormal vaginal bleeding that had persisted for a year. Three months prior to admission, on physical examination, a cauliflower-like tumor measuring 30 × 20 × 40 mm was found in the middle anterior vaginal wall. A colposcopy-guided biopsy revealed an adenocarcinoma of the vagina. One month later the patient underwent transurethral resection of a tumor in the mid-posterior urethral wall, and the pathology report also indicated an adenocarcinoma. Positron emission tomography/computed tomography (PET/CT) demonstrated urethral and vesical invasion with no distant metastasis. Furthermore, a cystoscopy indicated a cauliflower-like neoplasm about 30 mm in diameter in the bladder neck.

**Figure 2:**
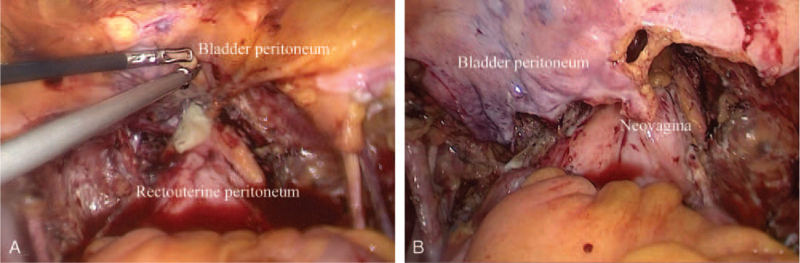
(A) The rectouterine peritoneum was sutured with the posterior margin of vulva. The free bladder peritoneum was sutured with the anterior margin of vulva. (B) Part of the bladder peritoneum was cut off. Vaginal reconstruction was performed using the partial bladder peritoneum as the anterior vaginal wall and the rectouterine peritoneum as the posterior wall.

The patient underwent an APE via a laparoscopic approach combined with a perineal approach. Radical hysterectomy with bilateral adnexectomy, pelvic lymphadenectomy, cystectomy with bladder peritoneum preservation, as well as resection of the vagina and urethra were performed. Subsequently, the rectouterine peritoneum was pulled out and sutured with the subcutaneous tissue and skin of posterior margin of vulva to form the posterior vaginal wall. The free bladder peritoneum was sutured with the anterior margin of vulva (Fig. 2A), and part of it was cut off to serve as the anterior vaginal wall. After colpoplasty by suturing the partial bladder peritoneum with the rectouterine peritoneum (Fig. 2B), the neovagina was approximately 10 cm in length with the vaginal orifice easily accommodating the size of one finger. The residual bladder peritoneum was sutured to the rectal fascia in order to create a mechanical support for the bowels. Finally, the patient underwent urinary reconstruction by ileal conduit formation and end-to-end ileoileostomy. The distal part of the neobladder was exteriorized as an output duct using an ileostomy at the right lower abdomen, with an indwelling de Pezzer catheter. A pelvic drainage tube was also placed. The whole operation lasted 300 minutes, with an estimated blood loss (EBL) of 400 mL and no intraoperative complications. The final pathological examination indicated clear cell adenocarcinoma of the vagina that invaded the urethra and the bladder. No metastatic lymph nodes were observed, and the resected margins were negative. The patient was diagnosed with stage IVA vaginal adenocarcinoma and was discharged on postoperative day 10, in the absence of adverse events. The patient was doing well at the 10-month follow-up visit.

### 3.2 Case 2

A 50-year-old woman was admitted to our department due to recurrence of stage IIB squamous cell carcinoma of the cervix after radiotherapy (94 Gy in 33 fractions) and 5-cycle of chemotherapy. Physical examination showed a cervical neoplasm measuring 30 × 20 mm with involvement of the upper half of the vaginal wall and parametrium. PET/CT confirmed a hypermetabolic focus at the cervix and absence of distant metastasis.

The patient underwent laparoscopic APE. There was tight adhesion between the bladder and the anterior wall of the uterus. Radical hysterectomy with bilateral adnexectomy, pelvic lymphadenectomy, intraperitoneal cystectomy, and partial resection of the urethra were performed. The bladder peritoneum was preserved and sutured to the rectal fascia to achieve closure of the pelvic cavity. Bilateral ureterocutaneostomy was performed with 2 mono-J catheters as ureteral stents, and a pelvic drainage tube was inserted. Samples were taken of enlarged lymph nodes that were suspicious for metastatic disease, and histological evaluation confirmed metastases in the right internal and left external iliac lymph nodes. This procedure lasted 320 minutes, with an EBL of 200 mL. The operative and postoperative courses were uneventful. The final pathological examination indicated poorly differentiated squamous cell carcinoma of the cervix invading the partial vaginal wall and bilateral parametrium. While there were 2 metastatic lymph nodes among the 4 left pelvic lymph nodes sampled, no invasion of the bladder was observed and the resection margins were negative. Finally, the patient was discharged on postoperative day 11. At the 14-month follow-up visit, the patient was doing well.

### 3.3 Case 3

The patient had an adenocarcinoma of the cervix and had undergone radiotherapy (60 Gy in 30 fractions) and 5-cycle of chemotherapy. On physical examination, the top of the vagina presented erosion-like changes and contact bleeding, with involvement of the upper half of the vaginal wall and left parametrium. Magnetic resonance imaging showed a mass measuring 21 × 23 × 28 mm, located predominantly in the anterior lip of the uterine cervix, with questionable invasion into the parametrium and the vagina. PET/CT demonstrated a cervical tumor without distant metastasis.

**Figure 3:**
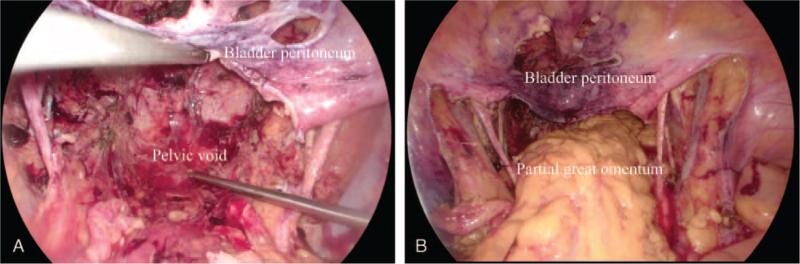
(A) The pelvic dead space was created after total pelvic exenteration. (B) A portion of the greater omentum was transposed into the pelvis to fill the dead space, and the bladder peritoneum was sutured to the pelvic floor fascia.

The patient underwent TPE during laparoscopic and transperineal surgeries. There was tight adhesion between the bladder and the anterior wall of the uterus and vagina. Radical hysterectomy, bilateral adnexectomy, pelvic lymphadenectomy, resection of the vagina, rectum, anus, bladder, and urethra were performed with bladder peritoneum preservation (Fig. 3A). The specimens were removed through the vulva before vulvalplasty. Afterwards, several reconstructive procedures were undertaken. A portion of the greater omentum was transposed into the pelvis to fill the dead space. The bladder peritoneal barrier was sutured to the pelvic floor fascia in order to separate the abdominal contents from the lower pelvic area (Fig. 3B). Sigmoidostomy and bilateral ureterocutaneostomy were performed, with 2 ureters supported by 2 mono-J catheters. A pelvic drainage tube was also inserted. The whole operation lasted 400 minutes, with an EBL of 600 mL, and no intraoperative complications were identified. Final pathological examination indicated poorly differentiated endometrioid adenocarcinoma of the cervix. The cervical tumor metastasized to the vagina, the left parametrium and the bladder. The primary and metastatic tumors were completely removed with the resected margins negative. In addition, no rectal invasion, and lymph node metastasis was observed. The patient had an uneventful postoperative course and was doing well at the 6-month follow-up visit.

## 4 Discussion

Since Alexander Brunschwig first introduced PE in 1948,^[1]^ advances in patient selection, preoperative preparation, surgical technique, and postoperative care have spared many patients from unnecessary complications; however, morbidity following PE still remains a concern.^[3]^

A substantial proportion of postoperative complications are associated with pelvic dead space after PE. The absence of mechanical support after the removal of the muscles of the pelvic floor and pelvic organs may allow intraabdominal contents to descend, which together with bowel adhesions deep in the denuded pelvis, may lead to intestinal obstruction. When postoperative radiotherapy is needed, the risk of chronic radiation enteritis increases. Moreover, small bowel translocation into the pelvis may also result in the occurrence of a perineal hernia. The void created after radical multivisceral resection leads to pelvic fluid accumulation and may increase the risk of perineal wound infection, abscess formation, and persistent discharge. In addition, the downward pressure of descending intraabdominal contents and the accumulation of fluid in the pelvis may contribute to wound dehiscence. This series of events has been termed the “empty pelvic syndrome” by some authors.^[4]^ Therefore, post-PE management of the pelvic dead space poses a challenge to gynecologists.

To minimize these unfavorable consequences caused by pelvic voids, a number of methods have been used for pelvic floor reconstruction after extirpation of pelvic organs; however, all these methods have advantages and disadvantages, but none has gained widespread acceptance. Some of these methods include artificial materials, such as polypropylene mesh,^[4]^ prosthetic implants,^[6]^ silicone expanders,^[14]^ and degradable mesh.^[4]^ Other methods employ autologous tissues, including the greater omentum,^[5]^ myocutaneous flaps based on the gracilis,^[7]^ rectus abdominis muscle,^[8]^ gluteus maximus,^[9]^ or the latissimus dorsi muscle.^[10]^

Pelvic exenteration is traditionally performed with an open approach. However, recent advances in medical technology make it possible to perform complex laparoscopic operations. Laparoscopic PE has been demonstrated to be a feasible procedure with many advantages over open PE, such as less EBL, a shorter duration of hospitalization, and lower postoperative complication rate.^[15,16]^ The above reconstructive techniques often require additional resources including time, cost, and expertise while negating some benefits of the laparoscopic approach.

This report describes a novel method that employs the preserved bladder peritoneum to close the pelvic void after laparoscopic APE, along with part of the greater omentum after TPE. To the best of our knowledge, this is the first report of the use of this approach.

The major strength of this procedure in our cases is that it provides a biological partition between abdominal contents and the pelvic cavity after PE, which can prevent the small bowel entering the lower pelvis. The smooth bladder peritoneum covering the rough pelvic wall can not only decrease the incidence of adhesions, but also absorb exudate present in the wound. The efficacy of using the greater omentum for pelvic floor reconstruction is due to its rich blood supply and lymphatic network, large surface area, and malleable volume, as confirmed by numerous studies.^[5]^ In addition, the use of the bladder peritoneum and the greater omentum has many common advantages. Compared to artificial materials, there is a reduction in allergic and immune reactions, and the risk of displacement is less. Compared to the use of other autologous tissues, the surgical technique presented here is less invasive and easier. There were also no scars on the body due to graft harvesting associated with donor-site morbidity, and it is convenient to peel the peritoneum off the bladder by laparoscopy. The data demonstrate that the newly formed pelvic floor covered the denuded pelvic cavity and prevented the remaining bowel from descending into the lower pelvis effectively.

PE has functional, psychological, and psychosexual influence on patients after the surgery; therefore, indications for PE should be carefully determined. The surgical procedures outlined in these cases are not applicable in certain instances, for example: in patients with peritoneal dissemination, distant visceral metastasis, or extrapelvic lymph node involvement. We carefully selected patients in this study, and these operations were performed successfully, achieving clear resection margins (R0). No intraoperative or postoperative complications were observed in any of the 3 patients. All patients were doing well at the most recent follow-up.

The main limitations of this study are the small number of cases and the relatively short follow-up period; hence, further investigations are still required. Nonetheless, these results demonstrate the short-term effectiveness and safety of this new approach in selected patients.

## 5 Conclusion

In conclusion, it is reasonable to utilize the preserved bladder peritoneum for closure of the pelvic cavity following laparoscopic PE, either with or without partial utilization of the greater omentum. Our method employs a simpler technique than other similar operations, and can reduce the risk of empty pelvic syndrome.

## Author contributions

**Conceptualization:** Ping Wang.

**Data curation:** Yiran Wang, Ping Wang.

**Supervision:** Ping Wang.

**Writing – original draft:** Yiran Wang.

**Writing – review & editing:** Yiran Wang, Ping Wang.
